# Network pharmacology identifies fisetin as a treatment for osteoporosis that activates the Wnt/β-catenin signaling pathway in BMSCs

**DOI:** 10.1186/s13018-023-03761-1

**Published:** 2023-04-22

**Authors:** Guihong Liang, Jinlong Zhao, Jianke Pan, Yuan Yang, Yaoxing Dou, Weiyi Yang, Lingfeng Zeng, Jun Liu

**Affiliations:** 1 The Second Clinical Medical College of Guangzhou University of Chinese Medicine, Guangzhou, 510405 China; 2 The Second Affiliated Hospital of Guangzhou University of Chinese Medicine (Guangdong Provincial Hospital of Chinese Medicine), Guangzhou, 510120 China; 3 The Research Team on Bone and Joint Degeneration and Injury of Guangdong Provincial Academy of Chinese Medical Sciences, Guangzhou, 510120 China; 4 The Fifth Clinical Medical College of Guangzhou University of Chinese Medicine, Guangzhou, 510405 China; 5 Guangdong Second Traditional Chinese Medicine Hospital (Guangdong Province Engineering Technology Research Institute of Traditional Chinese Medicine), Guangzhou, 510095 China

**Keywords:** Fisetin, Chinese traditional medicine, Osteoporosis, BMSCs, Mechanisms, Bone homeostasis

## Abstract

**Background:**

Although fisetin may exist widely in many natural herbs, its anti-OP mechanism is still unclear. The aim of this study is to explore the molecular anti-osteoporosis (OP) mechanism of fisetin based on network pharmacology and cell experiments.

**Methods:**

The target of fisetin was extracted by the Traditional Chinese Medicine Systems Pharmacology Database and Analysis Platform (TCMSP). The targets of OP were obtained by DisGeNET, GeneCards and the Comparative Toxicogenomics Database, and the targets of fisetin in OP were screened by cross-analysis. The protein–protein interaction (PPI) network was constructed by STRING, and the core targets were obtained. We performed gene ontology (GO) and Kyoto Encyclopedia of Genes and Genomes (KEGG) pathway enrichment analyses on common targets via the Database for Annotation, Visualization and Integrated Discovery. Finally, an in vitro cell experiment was used to verify the anti-OP effect and mechanism of fisetin.

**Results:**

There are 44 targets of fisetin related to the treatment of OP. The PPI results suggest that CTNNB1, CCND1, TP53, JUN, and AKT1 are the core targets. A total of 259 biological process, 57 molecular function and 26 cell component terms were obtained from GO enrichment analysis. The results of KEGG pathway enrichment analysis suggested that fisetin treatment of OP may be related to the Wnt signaling pathway, estrogen signaling pathway, PI3K-Akt signaling pathway and other signaling pathways. In vitro cell experiments showed that fisetin significantly increased the expression levels of ALP, collagen I, osteopontin and RUNX2 in bone marrow mesenchymal stem cells (BMSCs) (*p* < 0.05). Fisetin also increased the gene expression levels of Wnt3 and β-catenin (CTNNB1) in BMSCs, which indicates that fisetin can regulate the Wnt/β-catenin signaling pathway and promote the osteogenic differentiation of BMSCs.

**Conclusions:**

Fisetin acts on multiple targets and pathways in the treatment of OP; mechanistically, it regulates the Wnt/β-catenin signaling pathway, which promotes the osteogenic differentiation of BMSCs and maintains bone homeostasis. The results of this study provide a theoretical basis for further study on the complex anti-OP mechanism of fisetin.

## Introduction

Osteoporosis (OP) is a common systemic metabolic bone disease that is characterized by low bone mass and bone microstructure changes and easily leads to increased brittleness and fracture [1]. With the aging of the global population, OP will be more frequently diagnosed in middle-aged and elderly people. Some studies predict that women (50%) and men (20%) over the age of 50 will experience one or more OP fractures in their lives, which indicates that there will be great pressure on the medical system to develop strategies for the prevention and treatment of OP [2, 3]. Although the drugs currently used for the treatment of OP, including parathyroid hormone analogs, bisphosphonates and estrogen, are indeed effective, there may be various adverse reactions related to these drugs, which make their promotion and use far from ideal [4, 5]. Therefore, exploring safer and more effective therapies has great medical and social value.

**Fig. 1:**
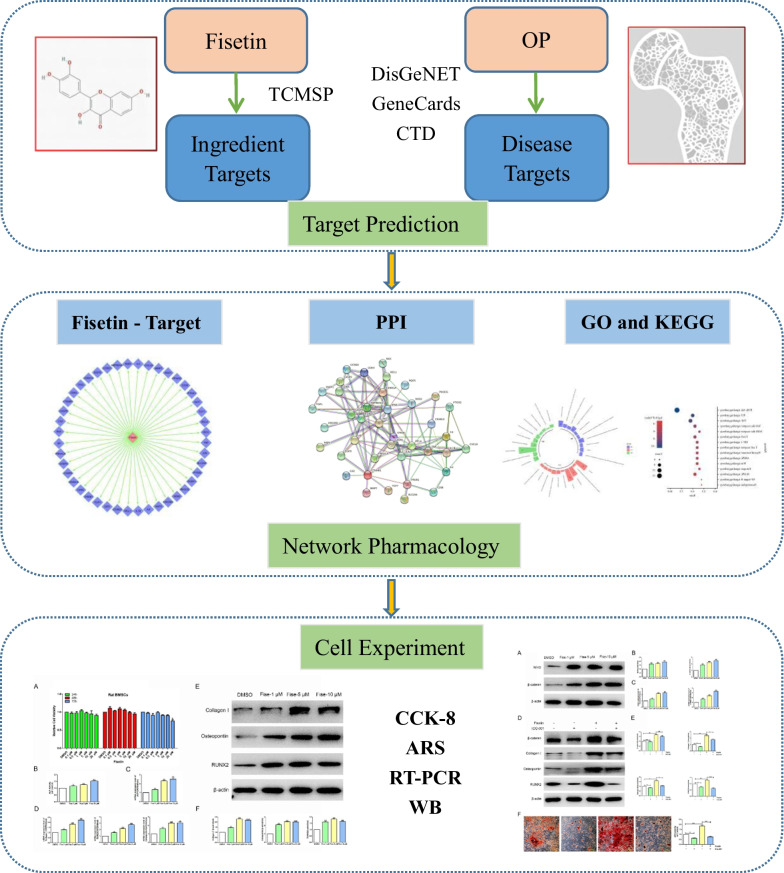
Overall flowchart of this study

OP belongs to the category of “*Gu Wei*” and “*Gu Bi*” in traditional Chinese medicine, and its pathogenesis is related to kidney deficiency. Therefore, the therapeutic mechanism of kidney tonifying on OP has become a hot spot in the field of traditional Chinese medicine. Chinese herbal medicines containing flavonoids are considered to tonify the kidney in traditional Chinese medicine. Fisetin is a flavonoid polyphenol compound that exists in a variety of Chinese herbal medicines, such as *Cotinus coggygria Scop.*, *Toxicodendron verniciflum*, and *Rhus chinensis Mill.* Flavonoids are thought to play an anti-OP role by regulating the balance of osteoblasts and osteoclasts [6]. Although fisetin may exist widely in many natural herbs, its anti-OP mechanism is still unclear, which will undoubtedly hinder the application of fisetin. Bone marrow mesenchymal stem cells (BMSCs) have great potential for osteogenic differentiation, and they are considered new therapeutic targets in the study of OP inhibition [7, 8]. According to Traditional Chinese medicine principles, the relationship between “*Shen Jing*” and BMSCs can be reflected in the process of inducing BMSC proliferation and differentiation, promoting their osteogenic differentiation and promoting bone formation by using kidney tonifying herbs. Based on the latest progress of the abovementioned theoretical and experimental research, this study will explore the biological mechanism of fisetin in the treatment of OP through network pharmacology combined with experimental verification (Fig. 1).

## Materials and methods

### Network pharmacology

#### Target of fisetin

Based on the Traditional Chinese Medicine Systems Pharmacology Database and Analysis Platform (TCMSP), the targets of fisetin were searched and screened. After obtaining the targets of fisetin, we uploaded the target to the UniProt protein database (https://www.UniProt.org) to obtain the corresponding standard target protein codes.

#### Target of OP

OP disease-related genes were obtained from GeneCards (https://www.genecards.org/), the Comparative Toxicogenomics Database (CTD) (http://ctdbase.org/) and the DisGeNET database (http://www.disgenet.org/web/DisGeNET/menu/home) with “osteoporosis” as the keyword. After obtaining the OP disease-related genes, the genes were mapped with the above fisetin action targets to reveal fisetin action targets for the treatment of OP. Mapping was implemented by Venny 2.1.

#### Construction of the network of fisetin and its predicted targets

Fisetin and predicted targets were imported into Cytoscape 3 7.1 software, and a network diagram of fisetin action targets was constructed.

#### Construction of the protein–protein interaction (PPI) network

The targets of fisetin obtained in “2.2” for the treatment of OP were uploaded to the STRING database (https://www.string-db.org/), and the protein interaction parameters were set for data analysis. The species parameter was set as “*Homo sapiens*”, and the confidence level was set as ≥ 0.90.

#### Gene Ontology (GO) analysis and KEGG pathway annotation

To clarify the biological processes and signaling pathways related to fisetin in the treatment of OP, we imported the potential targets into the Database for Annotation, Visualization, and Integrated Discovery (DAVID) (https://david.ncifcrf.gov/) for GO enrichment analysis and KEGG metabolic pathway analysis. We selected the results with the highest *p* values for visualization in the form of a circular bar graph and bubble graph.

### Experimental verification

#### Extraction, culture and identification of rat primary BMSCs

BMSCs were extracted from rats by whole bone marrow cell culture, and cell isolation, culture and identification were performed as previously described [9]. Briefly, the rats were euthanized and sterilized in 75% ethanol for 30 min, and the long bone shaft of the rats was isolated and collected. BMSCs were flushed out by an injection of Dulbecco’s modified Eagle’s medium (DMEM, C11885500BT, Gibco, USA) using a 20-mL syringe under sterile conditions. After centrifugation, the BMSCs were cultured in OriCell® rat bone marrow mesenchymal stem cell complete medium (batch No.: RAXMX-90011, Saiye (Suzhou) Biotechnology Co., Ltd., Suzhou, China). All of the abovementioned cells were cultured at 37 °C in an incubator containing 5% CO_2_.

#### Cell proliferation assay

BMSC viability was detected using the Cell Counting Kit-8 (CCK-8, C0038, Beyotime, China) assay following the manufacturer’s instructions. Specifically, BMSCs were plated at a density of 2500 cells per well in a 96-well plate. After 24 h of incubation, different concentrations (0.1 μM, 0.5 μM, 1 μM, 5 μM, 10 μM, and 20 μM) of fisetin (S2298, Selleck, USA) were added to the cells and incubated for 24, 48, and 72 h, respectively. Dimethyl sulfoxide (DMSO, D2650, Sigma, USA) was used as the control group. At the end of fisetin treatment, each well was subjected to 10 μL CCK-8 and incubated at 37 °C for 2 h. The absorbance of the optical density at 450 nm was determined using a microplate reader (M1000 Pro, Tecan, Switzerland).

#### Osteogenic differentiation determination and mineralization assessment

The surface of the 6-well plate for osteogenesis induction was coated with gelatin, and the BMSCs were plated at a density of 1 × 10^5^ cells per well. Osteogenically induced differentiation of the cells was performed using the Mesenchymal Stem Cell Osteogenic Differentiation Medium Kit (batch No.: RASMX-90021, Saiye (Suzhou) Biotechnology Co., Ltd., Suzhou, China) after reaching 70–80% confluence. For treatment, fisetin (10 μM) and/or ICG-001 (10 μM) were added to the osteogenic differentiation medium. The DMSO wells served as the control group. Mineralization of the calcium nodules was detected at day 30 of differentiation using alizarin red S (ARS) solution. Images were taken using an inverted microscope (ECLIPSE Ti2-E, Nikon, Japan). After that, 10% cetylpyridinium chloride was used to elute ARS staining, and the absorbance was detected at 562 nm using a microplate reader.

#### Alkaline phosphatase (ALP) activity assay

BMSCs were plated at a density of 1 × 10^5^ cells per well in a 6-well plate. The medium was replaced with osteogenic differentiation medium after 24 h. For treatment, fisetin at various concentrations (DMSO, 1 μM, 5 μM, and 10 μM) was added to the medium. The DMSO wells served as the control group. The ALP activity of cells was determined at day 10 of differentiation using an ALP Staining Kit (batch No.: P0321S, Beyotime Biotechnology, China). The absorbance at 405 nm for ALP was detected using a microplate reader.

#### Reverse transcription and real-time PCR (RT-PCR)

After rat BMSCs were treated with the different drugs, total RNA was extracted using TRIzol® Plus RNA Purification Kit (12183018A, Invitrogen, USA), and 1 μg of RNA from each sample was then reverse-transcribed to cDNA using the High Capacity cDNA Reverse Transcription Kit (4368814, Applied Biosystems, USA)) according to the manufacturer's protocol. For real-time PCR, ABI PrismTM 7500 (Thermo Life, USA) was performed using Fast SYBR@GREEN Master Mix (4385612, Applied Biosystems, USA) with the following primers. The primers used for ALP, Collagen I, RUNX2, Osteopontin, Wnt3, CTNNB1, and GAPDH amplifications are listed as follows: 5′-GGCGTCCATGAGCAGAACTACATC-3′ (ALP-Forward), and 5′-CAGGCACAGTGGTCAAGGTTGG-3′ (ALP-Reverse); 5′-TGTTGGTCCTGCTGGCAAGAATG-3′ (Collagen I-Forward), and 5′-GTCACCTTGTTCGCCTGTCTCAC-3′ (Collagen I-Reverse); 5′-CTTCGTCAGCGTCCTATCAGTTCC-3′ (RUNX2-Forward), and 5′-TCCATCAGCGTCAACACCATCATTC-3′ (RUNX2-Reverse); 5′-GACGATGATGACGACGACGATGAC-3′ (Osteopontin-Forward), and 5′-GTGTGCTGGCAGTGAAGGACTC-3′ (Osteopontin-Reverse); 5′-CAGCCTGACTTCCGAGCCATTG-3′ (Wnt3-Forward), and 5′-ACTCCCGATGCTTCTCCACCAC-3′ (Wnt3-Reverse); 5′-ACAAGCCACAGGACTACAAGAAACG-3′ (CTNNB1-Forward), and 5′-TCAGCAGTCTCATTCCAAGCCATTG-3′ (CTNNB1-Reverse); 5′-TCTCTGCTCCTCCCTGTTCT-3′ (GAPDH-Forward), and 5′-GTTCACACCGACCTTCACCA-3′ (GAPDH-Reverse). Relative gene expression was normalized to GAPDH as an internal standard, and the expression levels of target mRNAs were calculated by the 2^−△△CT^ method. Each assay was conducted in triplicate.

#### Western blotting (WB)

After BMSCs were treated with the different drugs, cell lysis buffer for WB and IP (P0013, Beyotime Biotechnology, China) was used to collect the total protein from the cells. The expression of relative proteins was detected by WB according to the protocol we described[1]. The primary protein antibodies were as follows: anti-RUNX2 (1:1000, 12556, CST), anti-Osteopontin (1:1000, ab63856, Abcam), anti-collagen I (1:1000, ab260043, Abcam), anti-β-actin (1:2000, 3700, CST), anti-β-catenin (1:1000, 8480, CST), and anti-Wnt3 (1:1000, ab32249, Abcam). HRP-conjugated goat anti-rabbit or anti-mouse secondary antibodies were used (7074, 7076, CST, USA).

#### Statistical analysis

The data are expressed as the mean ± standard deviation. T test was used for comparison between the two groups, and one-way ANOVA was used for multiple groups. *p* < 0.05 was considered to indicate statistical significance.

## Results

### Target of fisetin in the treatment of OP

**Fig. 2:**
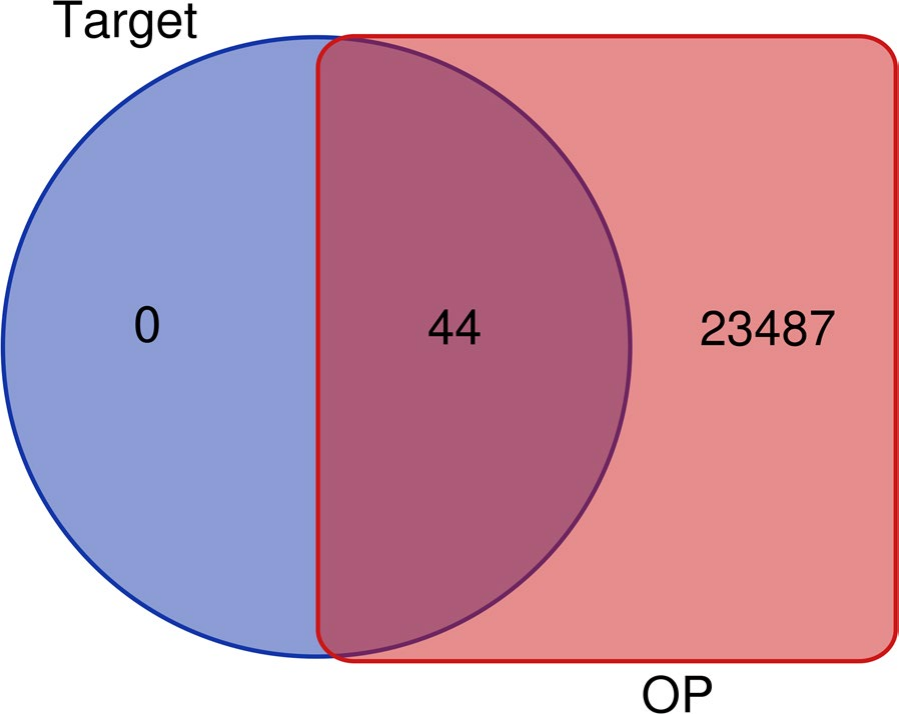
Venny diagram of fisetin targets and OP-related targets

A total of 46 potential therapeutic targets of fisetin (including 2 ineffective targets)were obtained from TCMSP. A total of 44 standardized target proteins were obtained by importing 46 potential therapeutic targets into the UniProt protein database. After repeat target removal of OP-related gene proteins in CTD, DisGeNET and GeneCards, a total of 23,531 OP targets were obtained. Venny 2.1 was used to map the fisetin targets and OP targets, and 44 common targets were obtained (Fig. 2).

### Fisetin–target network diagram

**Fig. 3:**
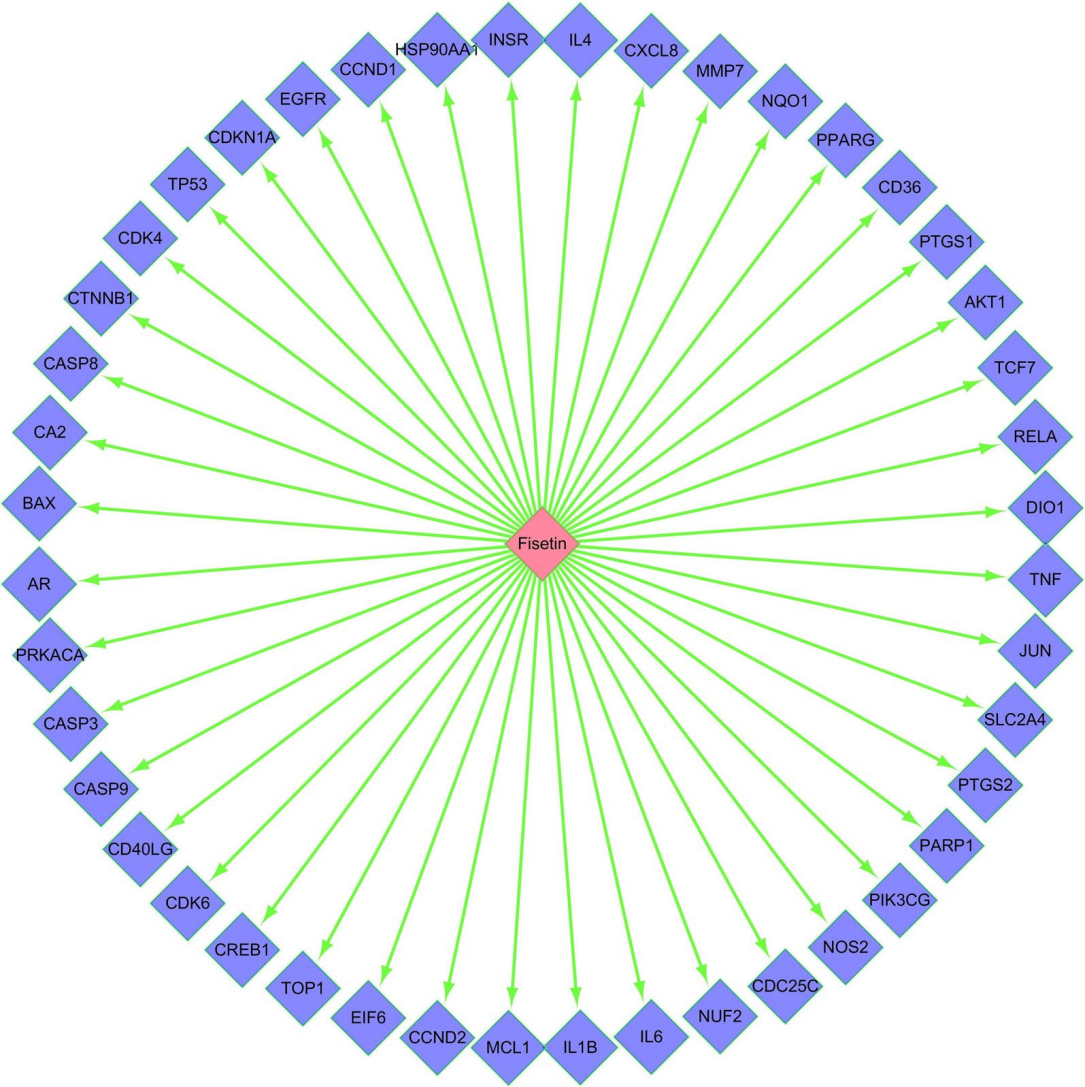
Potential target genes related to fisetin

The fisetin–target network diagram for OP was developed by Cytoscape 3 7.1 software (Fig. 3). From the network diagram, we can clearly see that fisetin acts on 44 target proteins, including CTNNB1, AR and JUN.

### PPI network and core target analysis

**Fig. 4:**
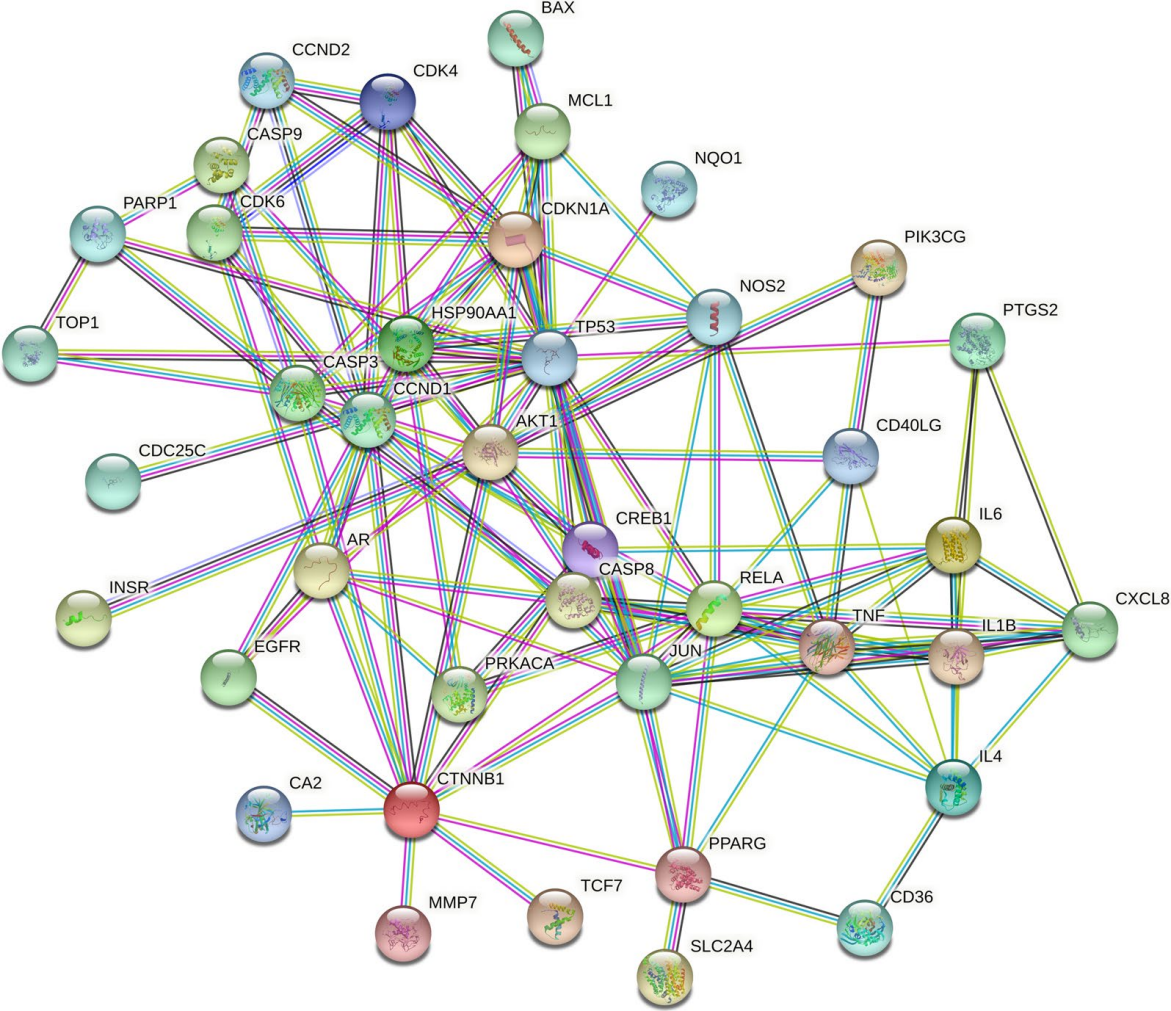
The PPI network of fisetin

**Fig. 5:**
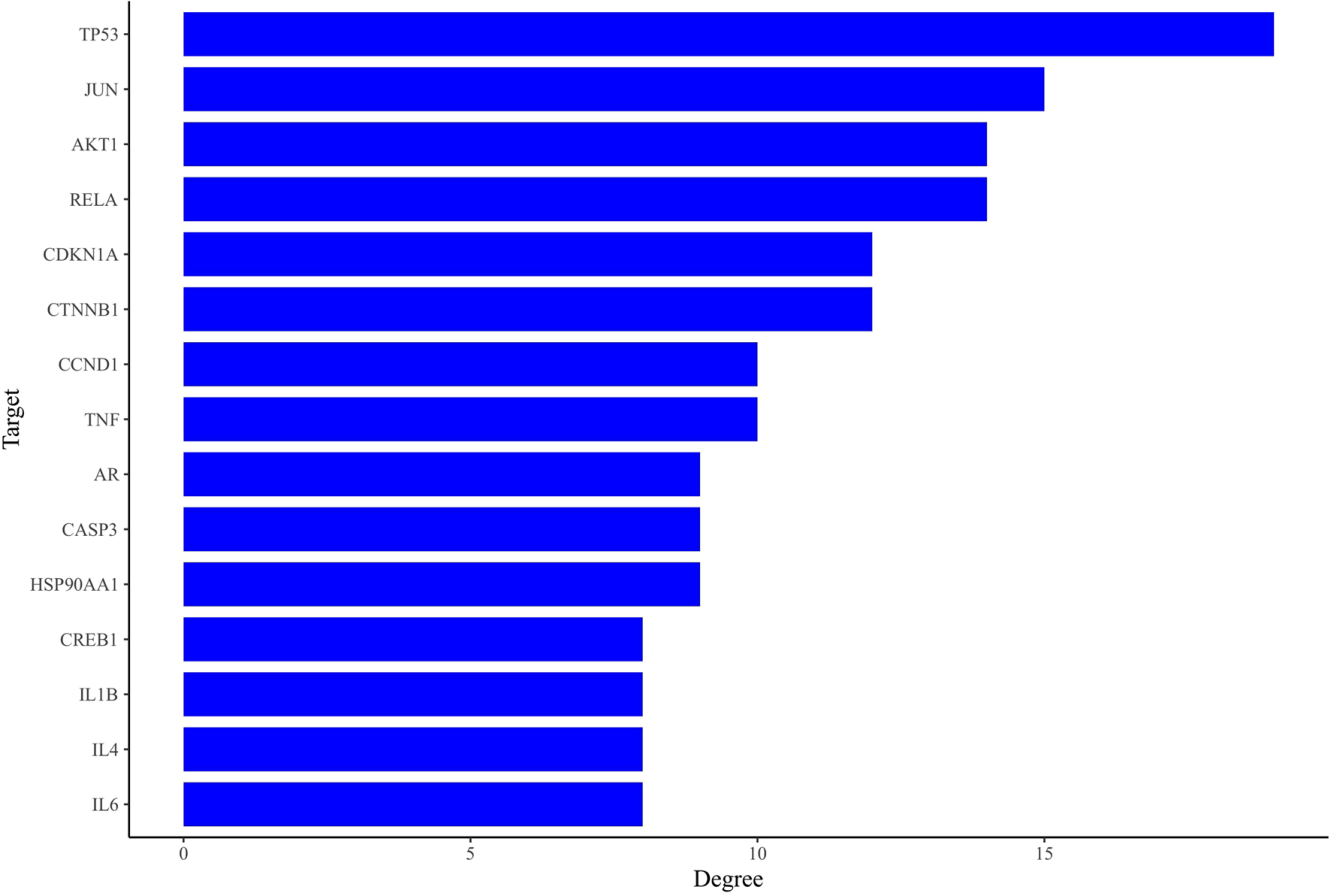
Histogram of the adjacent target number

The common gene targets of fisetin and OP were imported into the STRING database for analysis. The PPI results showed that 44 common targets participated in the network, and the average degree of the target was 5.77. The protein interaction diagram is shown in Fig. 4, from which we can see that the nodes are closely related. The top 15 targets in terms of degree values were TP53, JUN, AKT1, RELA, CDKN1A, CTNNB1, CCND1, TNF, AR, CASP3, HSP90AA1, CREB1, IL1B, IL4 and IL6 (Fig. 5).

### GO analysis and pathway annotation

**Fig. 6:**
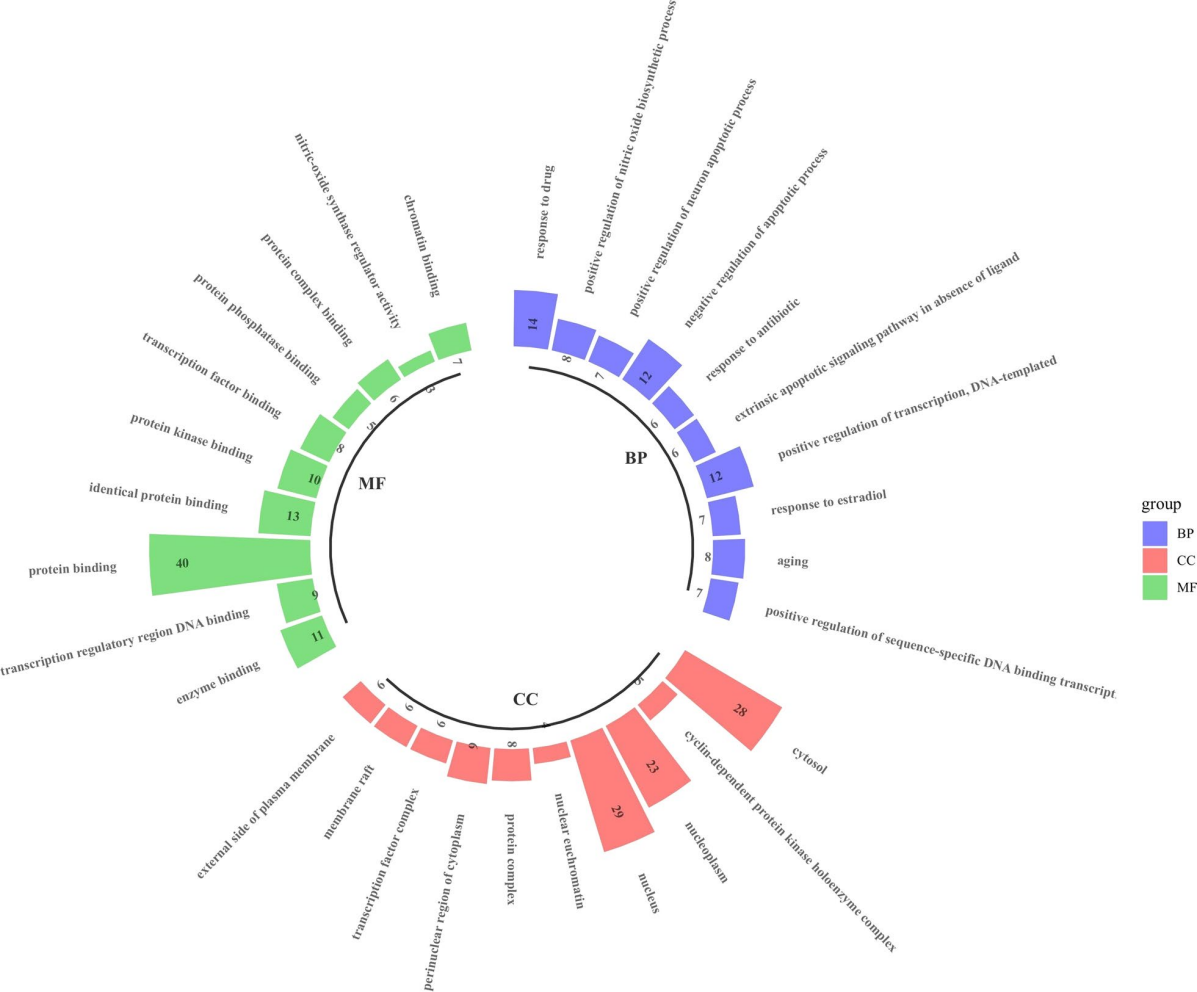
Go enrichment analysis for targets of fisetin against OP (TOP 10)

**Fig. 7:**
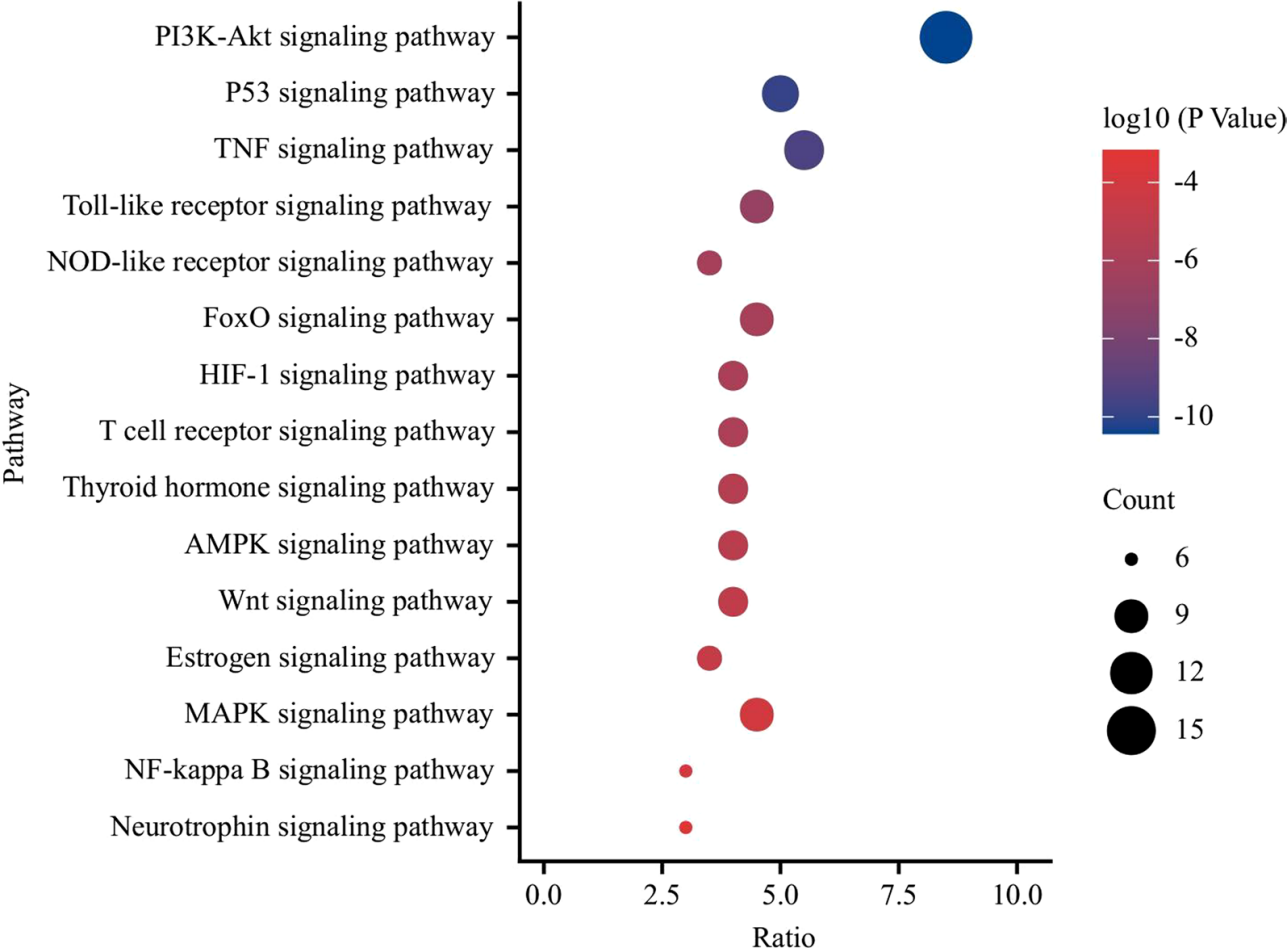
The bubble chart of KEGG pathway analysis (TOP 15)

A total of 259 biological process (BP), 57 molecular function (MF) and 26 cell component (CC) terms were obtained from the GO enrichment analysis. We show the top 10 most significant terms in each GO category with a circular bar graph (Fig. 6). The enrichment results of the KEGG pathway analysis suggested that there were 103 potential biological pathways related to fisetin in the treatment of OP; the top 15 signaling pathways with the strongest correlation with OP included the Wnt signaling pathway, estrogen signaling pathway, and PI3K-Akt signaling pathway (Fig. 7). These results suggest that fisetin plays an anti-OP role through a variety of biological and signaling pathways.

### Effects of fisetin on the cytotoxicity and osteogenic differentiation of BMSCs

**Fig. 8:**
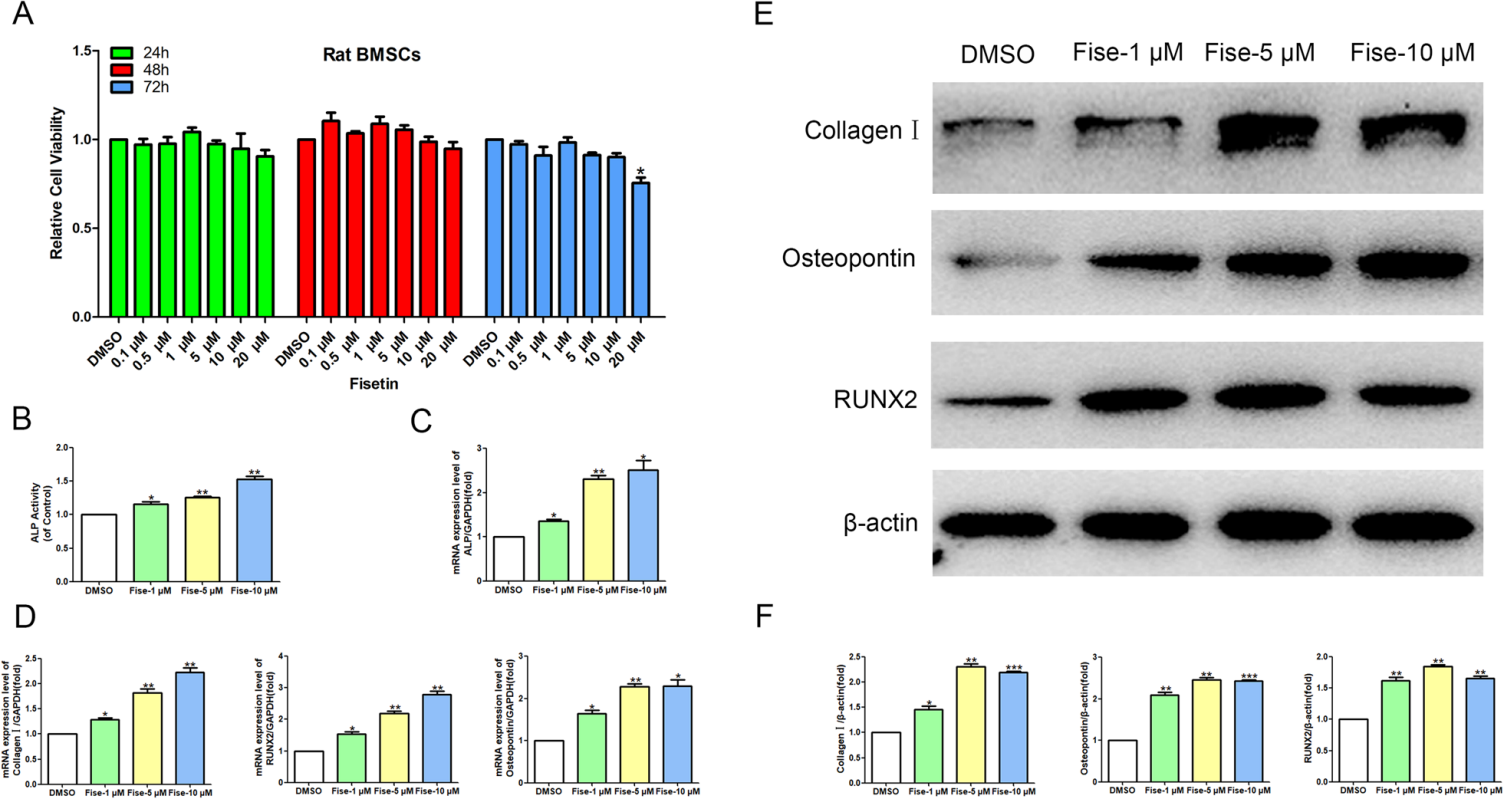
Fisetin promotes the osteogenic differentiation of BMSCs. **A** BMSCs were treated with increasing concentrations (0.1 μM, 0.5 μM, 1 μM, 5 μM, 10 μM, and 20 μM) of fisetin for 24, 48 and 72 h, followed by the determination of the OD value by CCK-8 assay. Cell viability was expressed as the mean ± SD from four independent experiments. Compared with the Concurrent DMSO group, *p < 0.05. **B** The ALP activity of BMSCs was expressed as the mean ± SD from three independent data points. Compared with the DMSO group, *p < 0.05; **p < 0.01. **C**, **D** BMSCs were treated with fisetin for 48 h, and mRNA expression was determined by RT–PCR. The relative mRNA expression of ALP, Collagen I, RUNX2 and Osteopontin was normalized to GAPDH. Compared with the DMSO group, *p < 0.05; **p < 0.01. **E** BMSCs were treated with fisetin for 48 h, followed by the analysis of collagen I, RUNX2 and osteopontin protein expression levels with WB. **F** Comparative analysis of grayscale values of relevant protein bands was performed, and the values were expressed as the mean ± SD from three independent experiments. Compared with the DMSO group, *p < 0.05; **p < 0.01; ***p < 0.001

To confirm the cytotoxic effect of fisetin on BMSCs, we treated BMSCs with increasing concentrations (0.1 μM, 0.5 μM, 1 μM, 5 μM, 10 μM, and 20 μM) of fisetin for 24, 48 and 72 h. The results show that 20 μM fisetin demonstrated significant cytotoxicity at 72 h but had no toxicity up to a concentration of 10 μM for 72 h (Fig. 8A).

Based on the above cytotoxicity results, BMSCs were cultured with 1, 5, and 10 μM fisetin for 48 h to detect the osteogenic differentiation of BMSCs. First, the ALP activity in BMSCs cultured with fisetin at concentrations of 1, 5 and 10 μM was significantly higher than that in the DMSO group (Fig. 8B). Second, the RT–PCR results showed that the mRNA expression levels of ALP, collagen I, osteopontin and RUNX2 in BMSCs cultured with fisetin at concentrations of 1, 5 and 10 μM were significantly higher than those in the DMSO group (Fig. 8C, D). Finally, the protein expression levels of collagen I, osteopontin and RUNX2 were all significantly increased in the fisetin treatment groups compared with the DMSO group (Fig. 8E, F).

### Effects of fisetin on the Wnt/β-catenin signaling pathway

**Fig. 9:**
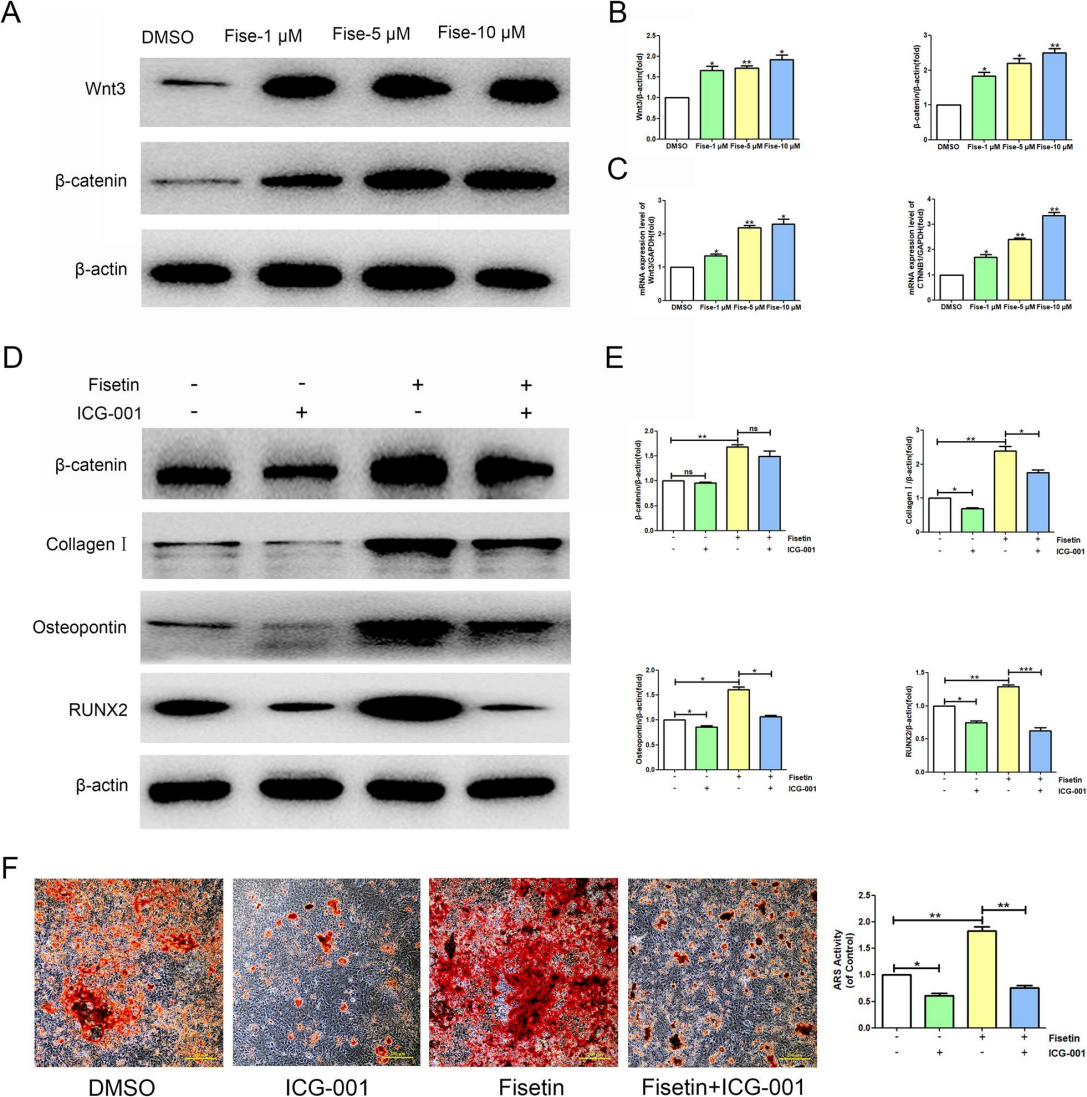
Fisetin promotes the osteogenic differentiation of BMSCs by activating the Wnt/β-catenin signaling pathway. **A** BMSCs were treated with increasing concentrations (1 μM, 5 μM, and 10 μM) of fisetin for 48 h, followed by the analysis of Wnt3 and β-catenin protein expression levels with WB. **B** Comparative analysis of grayscale values of relevant protein bands was performed, and the values were expressed as the mean ± SD from three independent experiments. Compared with the DMSO group, *p < 0.05; **p < 0.01. **C** BMSCs were treated with fisetin for 48 h, and mRNA expression was determined by RT–PCR. The relative mRNA expression of Wnt3 and CTNNB1 was normalized to GAPDH. Compared with the DMSO group, *p < 0.05; **p < 0.01. **D** BMSCs were incubated with or without 20 μM ICG-001 (S2662, Selleck, USA) and 10 μM fisetin for 48 h, followed by the analysis of collagen I, osteopontin, RUNX2, and β-catenin protein expression levels with WB. **E** Comparative analysis of grayscale values of relevant protein bands was performed, and the values were expressed as the mean ± SD from three independent experiments. ns* p* > 0.05, *p < 0.05; **p < 0.01; ***p < 0.001. **F** The mineralization capacity was measured by ARS staining and quantification (scale bars, 200 μm), *p < 0.05; **p < 0.01

The results of WB and RT–PCR showed that the protein and mRNA expression levels of Wnt3 and β-catenin (CTNNB1) in BMSCs cultured with fisetin at concentrations of 1, 5 and 10 μM were significantly higher than those in the DMSO group (Fig. 9A–C). To verify the role of the Wnt/β-catenin signaling pathway in the ability of fisetin to promote the osteogenic differentiation of BMSCs, we used the Wnt/β-catenin antagonist ICG-001 to inhibit Wnt-mediated transcription in BMSCs. The WB results showed that the expression levels of proteins related to osteogenic differentiation in BMSCs treated with 10 μM ICG-001 were significantly lower than those in the DMSO group. The protein expression levels of ALP, collagen I, osteopontin and RUNX2 in BMSCs cultured with both 10 μM ICG-001 and 10 μM fisetin were significantly lower than those in BMSCs cultured with 10 μM fisetin alone (Fig. 9D, E).

In addition, ARS staining verified the result. After 30 days of osteogenic differentiation induction, ARS staining indicated an increase in the stained area and number of mineralization nodules in the fisetin treatment groups compared with the DMSO group, and numbers of mineralization nodules in BMSCs cultured with both 10 μM ICG-001 and 10 μM fisetin were significantly lower than those in BMSCs cultured with 10 μM fisetin alone (Fig. 9F). Thus, all these data suggested that inhibiting the transcription of the Wnt/β-catenin signaling pathway reduces the ability of fisetin to promote the osteogenic differentiation of BMSCs, so fisetin may promote the osteogenic differentiation of BMSCs by regulating the Wnt/β-catenin signaling pathway.

## Discussion

OP affects the health of middle-aged and elderly people, and its prevalence and related medical costs are increasing. The active ingredients of Chinese herbal medicine are not only the active substances by which these compounds exert clinical efficacy but are also a resource for modern innovative drug development. Exploring safe and effective therapies to maintain bone homeostasis and promote bone remodeling is the main direction of OP prevention. Traditional Chinese medicine has an ancient history and certain advantages in the treatment of OP, and the application of traditional Chinese medicine can effectively maintain the dynamic balance between osteoblasts and osteoclasts [10, 11]. According to the theory of traditional Chinese medicine, OP belongs to the category of “*Gu Wei*”, “*Gu Ku*” and “*Gu Bi*”, and it is proposed that tonifying the kidney is the key to the treatment of OP. Fisetin is a flavonoid compound that has the potential effect of tonifying the kidney in the theory of traditional Chinese medicine. In this study, network pharmacology and cell experiments were combined to further study the biological mechanism of fisetin in the treatment of OP from the “disease–drug–target–pathway” perspective.

Understanding the bioactive pathway and regulatory mechanism of fisetin against OP at the molecular level is conducive to promoting the modernization of traditional Chinese medicine. Network pharmacology research revealed 44 overlapping targets for fisetin in the treatment of OP. Through PPI network interaction analysis, it was concluded that the core targets of fisetin in the treatment of OP are CTNNB1, CCND1, TP53, JUN, AKT1, RELA, CDKN1A, TNF, AR, etc., which indicates that fisetin plays a direct or indirect regulatory role in OP by acting on the above targets. GO and KEGG enrichment analyses of 44 common targets of fisetin and OP showed that fisetin affects multiple components, targets and pathways in the treatment of OP. Its mechanism of action is related to the Wnt signaling pathway, estrogen signaling pathway, PI3K-Akt signaling pathway and other signaling pathways. The coupling of bone formation and bone resorption is the basic process of bone metabolism. When the rate of bone resorption is greater than that of bone formation, OP will occur. The Wnt/β-catenin signaling pathway can not only regulate bone formation through osteoblasts but can also negatively regulate the differentiation of osteoclasts to maintain bone homeostasis, which plays an important role in the physiological and pathological processes of OP [12, 13]. BMSCs are a kind of adult stem cell that are highly plastic and have a wide range of sources. Under certain culture conditions, BMSCs can show strong proliferation and differentiation potential; for example, they can differentiate into mesodermal cells to form osteoblasts and muscle cells [14].

Recent studies have suggested that fisetin can improve the level of bone mineral density in ovariectomy-induced osteoporosis model rats [15] and promote osteogenesis in MC3T3-E1 cells and vertebrae formation in zebrafish larvae via the GSK-3β/β-catenin pathways [16]. However, the molecular mechanism of fisetin in osteoporosis remains unclear, especially its regulatory effect on BMSCs. To further explore the mechanism of fisetin in the prevention and treatment of osteoporosis, we investigated the effect of fisetin on the osteogenic differentiation of BMSCs by in vitro cell experiments. Our cell experiment results showed that the concentrations of 1, 5 and 10 μM fisetin could significantly increase the expression levels of ALP, collagen I, osteopontin and RNX2 in BMSCs, which indicated that fisetin could promote the osteogenic differentiation of BMSCs. We also found that fisetin regulates the CTNNB1 gene (encoding the protein β-catenin) and the Wnt signaling pathway through network pharmacological analysis. Therefore, we further explored the effect of fisetin on Wnt/β-catenin signaling pathway activity. Our experimental results show that fisetin can increase the gene expression levels of Wnt3 and β-catenin (CTNNB1) in BMSCs and then improve the vitality, proliferation, differentiation and mineralization of BMSCs, which indicates that fisetin can regulate the Wnt/β-catenin signaling pathway and promote the osteogenic differentiation of BMSCs.

## Conclusions

In conclusion, this study identified the core targets, signaling pathways and molecular mechanisms of fisetin in the treatment of OP through network pharmacological analysis. Further cell experiments confirmed that fisetin can significantly increase the expression levels of ALP, collagen I, osteopontin and RUNX2 in BMSCs, and it may exert its effect by regulating the Wnt/β-catenin signaling pathway to promote the osteogenic differentiation of BMSCs and maintains bone homeostasis. The results of this study enrich the understanding of the mechanism of fisetin in the treatment of OP, which provides a theoretical basis for the next step of new drug development and clinical application.

## Data Availability

All the necessary data supporting the result and conclusion of the study have been incorporated in the manuscript.

## Abbreviations

OP: Osteoporosis
TCMSP: Traditional Chinese Medicine Systems Pharmacology Database and Analysis Platform
CTD: Comparative Toxicogenomics Database
PPI: Protein–protein interaction
GO: Gene ontology
KEGG: Kyoto Encyclopedia of Genes and Genomes
DAVID: Database for Annotation, Visualization and Integrated Discovery
BMSCs: Bone marrow mesenchymal stem cells
ARS: Alizarin red S
BP: Biological process
MF: Molecular function
CC: Cell component
